# Antiseptics in Obstetric Nursing
*Antiseptics in Obstetric Nursing. By John Shaw, M.D. London: H. K. Lewis.


**Published:** 1891-07-25

**Authors:** 


### Turning Over New Leaves.

### Antiseptics in Obstetric Nursing.*

One of the most striking and beneficent of all the advances that have been made in modern medicine is the discovery of the part played by disease germs, and of certain effectual methods of checking and counteracting the deadly influence of those germs. To the well-instructed obstetric physician and nurse the possibilities of certain diseases at the time of parturition are perfectly clear; the causes and conditions of them are known and understood, the method of prevention is also quite familiar, and the course of the disease and the mode of cure, in cases where early discovery makes cure possible, are both plain sailing. Puerperal fever has to a large extent been robbed of its terrors. No department in medical practice gives more satisfactory results at the present day than obstetrics; and there are no cases to which the skilled nurse is called that better repay the exercise of trained skill

The results which we have outlined are due to what may be called the antiseptic system of nursing and treatment. The clear understanding of this system and the thorough practice of it in detail prevent more otherwise inevitable disease and suffering, and thus save more lives than any other discovery known to modern medicine. It is of prime importance, therefore, that the antiseptic system of nursing and treatment be clearly expounded as to its principles, and as to the reasoned and experimental foundations on which it rests, and also intelligently taught in practical details. Dr. John Shaw has brought together in a book of a little more than a hundred pages, all the essential principles and details of antiseptic obstetric practice, and antiseptic nursing. Dr. Shaw gives his book the modest title of "Antiseptics in Obstetric Nursing." The book is written by a man who has thoroughly mastered his subject in theory, and who has evidently been in the constant habit of applying his principles in practice. It is intelligent and conscientious. It gives both the "How" and the "Why" of obstetric nursing. All nurses should understand the "why" as well as the "how," and they will understand both the one and the other if they carefully read and master Dr. Shaw's book. We congratulate the author on the production of a scientific, practical, and most useful manual.

* Antiseptics in Obstetric Nursing. By John Shaw, M.D. London: H. K. Lewis.

